# Effect of weight status on pregnancy outcome in intra cytoplasmic sperm injection

**Published:** 2013-09

**Authors:** Rehana Rehman, Zahir Hussain, Syeda Sadia Fatima

**Affiliations:** 1 *Department of Physiology, Bahria University Medical and Dental College, Karachi, Pakistan.*; 2 *Department of Physiology, Umm Al-Qura University, Makkah, Kingdom Saudia Arabia.*; 3 *Department of Physiology, University of Karachi, Karachi, Pakistan.*; 4 *Department of Biological and Biomedical Sciences, Aga Khan University, Karachi, Pakistan.*

**Keywords:** Intracytoplasmic sperm injection, Body mass index, Controlled ovarian stimulation, Gonadotrophin -releasing hormone agonists, Embryo transfer.

## Abstract

**Background:** There has been an increase in number of obese infertile females booked for advanced infertility treatment procedures like in vitro fertilization (IVF) and intra cytoplasmic sperm injection (ICSI). The knowledge of impact of body mass index (BMI) on reproductive outcome can help to counsel these patients.

**Objective: **To compare reproductive outcome in females of different BMI after ICSI.

**Materials and Methods:** Cross-sectional study of 323 females was conducted from June 2010 till August 2011. Females were grouped on the basis of BMI; underweight, (BMI <18 kg/m^2^), normal weight, (BMI 18-22.9 kg/m^2^) overweight (BMI 23-25.9 kg/m^2^) and obese (BMI ≥26 kg/m^2^). The procedure involved down regulation of ovaries, controlled ovarian stimulation, ovulation induction by hCG, oocyte pickup, in vitro fertilization and embryo transfer of blastocysts. The oocyte yield and embryological data of all BMI groups was compared by ANOVA (analysis of variance). Pregnancy outcome of these was categorized as; no conception βhCG <5 m IU/ml, preclinical abortion with βhCG >5 m IU/ml, no cardiac activity on trans vaginal scan (TVS) and clinical pregnancy with βhCG >5mIU/ml and cardiac activity on trans vaginal scan.

**Results: **Females with BMI 23-25.99 kg/m^2^ had maximum oocyte retrieval, fertilization, implantation and clinical pregnancy rates in comparison to obese females with BMI ≥26 kg/m^2^.

**Conclusion:** A BMI cut off value of above 26 kg/m^2^ in our study population is associated with a negative impact on pregnancy outcome.

## Introduction

Obesity is a rapidly growing worldwide phenomenon; with an incidence of 12% in women of child bearing age in Western Europe and 25% in North America (1-4). The pattern of obesity is currently emerging as an epidemic. In addition to diabetes, hypertension and cardiovascular diseases, obesity leads to disturbances in wide spectrum of reproductive dysfunctions ranging from an-ovulatory cycles, delay in conception, high rates of miscarriages, gestational diabetes, preeclampsia, and high neonatal morbidity and mortality rates (5, 6). 

Fertility is the ability to give birth to babies whereas fecundity expresses the monthly probability of reproduction in a woman (7). The chance of spontaneous conception decreases in sub fertile, normal ovulatory women by 5% for each unit increase in body mass index (BMI) (8). Infertility is attributed to overweight and obese women as a result of reproductive impairments occurring at many levels of the hypothalamic-ovarian-uterine axis, interruption to which results in ovulatory dysfunction (oligo-/an ovulation) as well as menstrual disturbances (9, 10). 

These impairments lead to delay in natural conception, increased referral to assisted reproductive clinics and fewer conception results, after reproductive treatment protocols (11-15). The use of assisted fertility techniques and treatment procedures has also been linked to the rising rates of obesity which in turn is expected to be a potential cause for an increase in sub fertility (15). The reproductive treatment protocols for couples in whom pregnancy fails to occur by ovulation induction and intrauterine insemination are; in vitro fertilization (IVF) or intra cytoplasm sperm injection (ICSI), which aids in the introduction of a single sperm in the ooplasm. The impact of obesity on duration of stimulation, number of oocytes, oocyte maturity, implantation and clinical pregnancy rates can be assessed by change in BMI defined as weight in kilograms divided by height in meters squared (kg/m^2^) (16, 17).

Shah *et al* suggested that obesity may affect results of treatment procedures both at the level of the ovary as well as after embryo transfer in terms of impaired endometrial lining (18). High BMI has been shown to be associated with lower success rates following assisted reproduction, including the need for prolonged ovarian stimulation and high doses of gonadotrophins (19, 20). With the increasing prevalence of obesity in reproductive age group, more women look forward for infertility treatment and contradictory reports on reproductive outcome. Hence it is imperative to assess the impact of obesity on IVF/ICSI outcomes to better counsel these women. Keeping this in view, the objective of this study was to assess the effect of BMI on reproductive outcome of ICSI procedures and to determine the BMI cut off values in Pakistani population for favorable pregnancy outcomes. 

## Materials and methods

The research was conducted in Islamabad Clinic serving infertile couples at Saudi Pak Tower from June 2010 till August 2011. In this study, we recruited total of 323 couples. Females between the age of 18-41 yr, duration of infertility >2 years, having normal ovulatory cycles (25-35 days), a basal follicle stimulating hormone (FSH) serum level <10 m IU/mL, and with no known ovarian morphological abnormalities, were included in this study. The long protocol of gonadotrophin releasing hormone (GnRH) agonist down regulation, stimulation with injection of recombinant follicle stimulating hormone (rFSH; Puregon) and progesterone support with 400 mg cyclogest pessaries daily were administered.

Females with age greater than 41 years, a basal FSH serum level >10 m IU/ml, presence of poly cystic ovaries based on the presence of two of the following three criteria: oligo or an ovulatory cycles, ultrasound visualization of polycysts and clinical or biochemical evidence of hyperandrogenism, GnRh antagonist therapy, short down regulation with GnRH agonist, ICSI with sperm retrieval by testicular biopsy and frozen embryo transfers were excluded from this study (21). 

**Ethical statement**

All research protocol was approved by the Board of Advanced Studies and Research (BASR), University of Karachi No.0435/SC and Islamabad Clinic serving infertile couples at Saudi Pak Tower No.11/R- ICSI. A written informed consent was obtained from all participants.

**Measurement of blood pressure**

A single blood pressure measurement, by the recommended procedure mainly on the left arm, with appropriate cuff of a standard mercury sphygmomanometer was taken and record was maintained. The staff was instructed to follow strict regulations with all precautions; respondent should remain attached, and hold the monitoring device on the upper arm at level of heart against his/her chest

**Anthropometric measurements**

Subjects were weighed on a digital weighing scale in kilogram with an accuracy of ±100 gr in their normal clothing without shoes. Standing body height (BH) was measured without shoes to the nearest 0.5 cm with the help of height scale (floor type ZT-120 EVERICH, China) with the shoulders in relaxed position and arms hanging freely. BMI was calculated as body weight in kilograms (kg) divided by the square of the body height in meters (m^2^) (22). Study subjects were grouped as per the WHO BMI classification for south Asian population as Group I: BMI <18(underweight), Group II BMI 18-22.9 (normal weight), Group III BMI 23-25.9 (overweight) and Group IV BMI ≥26 (obese) (23).

**Investigation protocol**

The study subjects were administered daily sub cutaneous (S/C) injection gonadotrophin releasing hormone agonist (Deca Peptyl 3.75 mg, Ferring) from day 21 of previous cycle followed by controlled ovarian stimulation (COS) by gonadotropin (Inj Puregon®, N.V. Organon, Oss, The Netherlands) sub cutaneous from 2^nd^-3^rd^ day of cycle till the administration of human chorionic gonadotropin (hCG). Maturity of follicle (20 mm) was assessed by series of transvaginal scan (TVS) started from 5^th^ day of COS followed by ovulation induction (OI) with intra muscular injection of hCG 10,000 I.U (Profasi®, Serono, Switzerland).

In oocytes pick up (OPU) eggs were retrieved 36 hours after OI by vaginal ultrasound probe with 16G adapter and double lumen oocyte aspiration needle on 14^th^, 15^th^ or 16^th^ day of COS. All collected eggs were treated and then transferred to the incubator for about 1-2 hours prior to insemination by ICSI procedures. Semen analysis performed by strict Kruger’s criteria and film was prepared by Silselect gradient. ICSI by micro injections of spermatozoa was performed at right angles to the position of polar body under the microscope. Fertilized embryos (presence of two pronuclei; 2PN) were assessed for cleavage and differentiation into distinct cell types with formation of fluid filled cavity (blastocysts). Embryo transfer (ET) of blastocysts was done five days after OI by Sims-Wallace Embryo Replacement Catheter under ultrasound guidance. Luteal support was maintained by progesterone vaginal pessaries (Cyclogest 400 mg) twice a day from the day of OPU (24). 

Single serum βhCG measurement was performed on specimens obtained by peripheral venipuncture 14 days after egg collection as the outcome marker. TVS was performed at 5 weeks gestation (22 to 32 days after fertilization) to identify clinical pregnancy from preclinical abortion (25). On the basis of βhCG and TVS, results were analyzed as non-pregnant (βhCG <5m IU/ml), preclinical abortions or biochemical pregnancy (βhCG >5m IU ml without any cardiac activity) and clinical pregnancy with βhCG >5m IU/ml with any cardiac activity (26).

**Statistical analysis**

Statistical comparison of all BMI groups was performed by using one way analysis of variance (ANOVA) via SPSS (version 15; SPSS Inc., Chicago, IL, USA). Clinical characteristics were summarized in terms of frequencies and percentages for qualitative variables (age group), mean±SD for continuous/quantitative variables. In all statistical analysis only p<0.05 was to be considered significant. Percentages of not pregnant, preclinical abortion and clinical pregnancy cases were tabulated for all BMI groups and compared by pair analysis Fertilization rate was defined as the proportion of oocytes resulting in two pronuclei formation (26). Mean implantation rate was the proportion of embryos transferred resulting in an intrauterine gestational sac. A clinical pregnancy defined by the presence of one or more gestation sacs by ultrasound (27). Pearson and or spearman correlation were applied to compare cleavage, fertilization and implantation rates of all groups of BMI where applicable. 

## Results

The detailed results are shown in Tables I-IV. Briefly, out of 323 participants, 41 results are not included; 14 females (34%) failed to respond while in 27 (66%) patients embryos were transferred before blastocysts maturation. Results of 282 showed that 21(7%) females were underweight with BMI <18 kg/m^2^, 78 (28%) were normal weight with BMI 18-22.9 kg/m^2^, 56 (20%) were overweight with BMI 23-25.99 kg/m^2^ and 127 (45%) were obese with BMI ≥26 kg/m^2^. Non-significant changes were observed in terms of infertility duration, where the maximum duration of five years was reported in obese females (54%) as compared to the other groups. 

The total number of successful clinical pregnancies in BMI <18 kg/m^2^ was 10 (48%), BMI 18-22.9 kg/m^2^ was 29 (37%), BMI 23-25.99 kg/m^2^ was 26 (46%) and BMI ≥26 kg/m^2^ was 36 (28%) however these results were not significantly different (Table I). Significant difference were seen in non-pregnant females 68 (53%) with BMI ≥26 kg/m^2^ as compared to 15 (27%) with BMI 23-25.99 kg/m^2^ (p=0.03), while non-significant results were seen in BMI range <18 and 18-22.9 kg/m^2^ (Table I). Clinical pregnancy was significantly high in BMI 23-25.99 kg/m^2^ group as compared to obese BMI ≥26 kg/m^2^ (p=0.017). The baseline characteristics of study subjects are shown in Table II. 

Comparison of oocyte yield, duration of treatment, drug used and details of embryological data (Table III) does not show any significant results except duration of stimulation that was found to be significantly higher with increasing BMI (p=0.027). Duration of stimulation was maximum, OPU on day 15 (60; 47%) in BMI ≥26 kg/m^2^. Oocyte recovery, fertilization and cleavage rate was positively correlated with BMI 23-25.99 kg/m^2^, whereas implantation rate and oocyte retrieval rate had an inverse correlation with BMI <18 kg/m^2^ (Table IV).

**Table I:** Comparison of reproductive outcome of ICSI in female groups with varying weight status

	**%ages**				**p (pair comparison)**					
**Reproductive Outcome**	**Under weight** **(<18)**	**Normal weight** **(18-22.9)**	**Over weight** **(23-25.99)**	**Obese** **(>26)**	**Under weight** **(<18)**			**Normal weight** **(18-22.9)**		**Over weight** **(23-25.99)**
					**Normal** **(18-22.9)**	**Over** **(23-25.99)**	**Obese** **(>26)**	**Over** **(23-25.99)**	**Obese** **(>26)**	**Obese** **(>26)**
Not pregnant	7 (33%)	30(38%)	15 (27%)	68 (54%)	0.666	0.571	0.086	0.158	0.036	0.001
Preclinical abortion	4 (19%)	19(24%)	15 (27%)	23 (18%)	0.609	0.483	0.918	0.750	0.282	0.182
Clinical pregnancies	10(48%)	29(37%)	26 (46%)	36 (28%)	0.385	0.926	0.077	0.283	0.187	0.017

**Table II:** Base-line clinical and physiological characteristics according to body mass index

**Variables**	**Underweight** **BMI <18**	**Normal** **BMI 18-22.9**	**Overweight** **BMI 23-25.99**	**Obese** **BMI ≥26**	**p-value**
Number	21	78	56	127	
Female age (years)	33.095 ± 1.11	32.167 ± 0.52	32.161 ± 0.666	31.89 ± 0.4	0.74
Age at marriage (years)	25.81 ± 1.453	24.949 ± 0.508	24.214 ± 0.57	25.248 ± 0.372	0.42
Age of menarche (years)	13.857 ± 0.261	14.038 ± 0.141	13.964 ± 0.157	14.134 ± 0.1	0.67
Duration of infertility	7.286 ± 0.799	7.218 ± 0.418	7.946 ± 0.634	6.642 ± 0.315	0.20
Systolic blood pressure (mm Hg)	122.048 ± 0.965	121.205 ± 0.492	121 ± 0.727	119.819 ± 0.464	0.09
Diastolic blood pressure(mm Hg)	73.857 ± 0.591	75.333 ± 0.403	75.089 ± 0.538	76.701 ± 0.367	0.001*

Values are represented as Mean±SD.

Differences among groups were assessed by using analysis of variance. Significance level at <0.05.

**Table III:** Response to ovarian stimulation and embryological data according to body mass index (BMI)

**Variable**	**Underweight ** **BMI <18**	**Normal** **BMI 18-22.9**	**Overweight** **BMI 23-25.99**	**Obese** **BMI ≥26**	**p-value**
Number	21	78	56	127	
Total number of puregons	54.821± 0.704	57.323 ± 0.914	55.905 ± 1	58.102± 0.966	0.28
No of puregons in one day	3.964 ± 0.087	4.05 ± 0.078	3.936 ± 0.074	4.014 ± 0.066	0.79
Follicle at ultrasound	20.667 ± 1.522	18.859 ± 1.05	21.679 ± 1.165	19.339 ± 0.76	0.25
Endo. lining	9.524 ± 0.94	8.167 ± 0.393	8.732 ± 0.44	8.646 ± 0.29	0.40
No of oocytes/patient	20.286± 1.543	18.449 ± 1.048	21.25 ± 1.147	18.921± 0.767	0.25
Oocyte retrieval rate (%)	98	97	98	97	0.72
No of Metaphase II oocytes	16.81 ± 1.656	14.833 ± 1.038	17.393 ± 1.169	14.961 ± 0.701	0.22
No of 2PN oocytes	16 ± 1.551	14.346 ± 1.005	17.036 ± 1.155	14.504 ± 0.7	0.20
Fertilization rate (%)	77	76	79	76	0.81
No of cleaved embryos	10.762 ± 1.327	10.167 ± 0.857	12.339 ± 0.941	10 ± 0.604	0.20
Cleavage rate (%)	51	52	57	52	0.58
No of transferred blastocysts	1.571 ± 0.111	1.654 ± 0.065	1.696 ± 0.076	1.591 ± 0.054	0.65
Number of gestational sacs	0.619 ± 0.161	0.447 ± 0.08	0.636 ± 0.111	0.37 ± 0.058	0.09
Implantation rate (%)	43	26	34	23	0.14

Values are represented as Mean±SD.

Differences among groups were assessed by using analysis of variance. Significance level at <0.05.

**Table IV:** Correlation of BMI with rates of reproductive outcome

	**Underweight (BMI<18)**		**Normal (BMI 18-22.9)**		**Overweight (BMI 23-25.99)**		**Obese (BMI ≥26)**	
	**r**	**p-value**	**r**	**p-value**	**r**	**p-value**	**r**	**p-value**
Fertilization rate	0.121	0.601	0.090	0.431	0.259	0.054	-0.013	0.88
Implantation rate	-0.394	0.077	-0.122	0.286	0.072	0.599	-0.147	0.09
Oocyte retrieval rate	-0.155	0.502	-0.122	0.286	-0.034	0.806	-0.050	0.57
Cleavage rate	-0.127	0.583	-0.109	0.344	0.266	0.048	0.006	0.94
Oocyte maturity rate	-0.081	0.727	0.122	0.289	0.220	0.104	0.094	0.29

Correlation applied by Pearson correlation coefficient.

## Discussion

Obese females are prone to have lower clinical pregnancy rates, and lower live birth rates as compared with women of normal BMI. This may be due to lack of up regulation of gonadotrophin receptors and steroid genesis in the ovary (18, 27). Gonadotrophin requirements are found to be higher in obese women (BMI>30 kg/m^2^) as compared to non-obese women with high risk of cycle cancellation due to poor ovarian response (28). This can be explained on the basis of reduction in delivery of hCG to the follicles required for ultimate oocyte maturation in obese women (29).

In our study, daily dose and total number of ampoules used for possible follicular maturation were more in obese females (BMI ≥26 kg/m^2^) though results were not significant. The results are supported by Esinler *et al* who found obesity as an independent risk factor for impaired oocyte maturation which calls for higher total doses of gonadotrophin stimulation in obese women (30). Highest number of oocyte, metaphase II oocytes and oocyte recovery rate retrieval was observed in female group with BMI 23-25.99 kg/m^2^ in our study. Wittemer *et al* found a positive correlation of BMI with number of stimulated follicles and significant negative correlation with ampoules of gonadotropins used and days of stimulation (31). 

Other studies documented fewer oocytes in obese women with BMI >25 kg/m^2^ in comparison with those with BMI <25 kg/m^2^ and fewer oocyte-cumulus complexes and retrieved metaphase II oocytes in women with BMI ≥30 kg/m^2^ (29, 30). In our study fertilization rate, cleavage rate and implantation rate of embryos was observed the most in BMI group 23-25.99 kg/m^2^ which is supported by low fertilization rates in women with (25<BMI<30 kg/m^2^) by another study (32). Implantation of fertilized ovum after ET is attributed to a dialogue between invading fertilized ovum and receptive endometrium. 

Some authors have identiﬁed a reduction in implantation rates among the obese women whereas others have not demonstrated a weight related reduction (20, 33-36). Women of BMI >25 kg/m^2^ were found to have a lower chance of implantation as compared to those with BMI 20-25 kg/m^2^ (37). As far as quality of embryo is concerned, some authors have reported a reduction in the overall quality of the embryos derived in an IVF cycle among higher BMI groups (16, 20, 30, 36). While another retrospective study concluded that the embryo quality was not impaired in overweight and obese women (35). 

In the feto maternal cross talk synchrony of blastocysts invasion with receptive endometrium is responsible for successful outcomes in ICSI. Since we selected blastocysts in our study to utilize embryos of superior developmental and highest implantation potential, the difference in implantation rates is largely attributed to difference in endometrial receptivity (38). Our data revealed an inverse correlation between oocyte yield and BMI while the effects of BMI on endometrial lining were not significant. The work done by Sathya *et al* and Dokras *et al *documented that BMI does not influence endometrial thickness, oocyte number, quality, implantation and pregnancy rates (10, 39). 

Few researchers suggested that with an increase in BMI (BMI ≥25kg/m^2^), amount of gonadotrophin and days of stimulation increase, while number of follicles decrease with decrease in pregnancy rates (37). In our study, clinical pregnancy rates were significantly lower in obese groups as compared with the rest of BMI groups. It has been a long argument to defer fertility treatment to overweight and obese women keeping in mind the cost, poor chances of success, higher risks of pregnancy loss and perinatal complications (40). 

Yet, the literature on the costs of fertility treatment, antenatal and peri partum care in obese women is not adequate. It has been established by our study, that obesity exerts its effect on reproductive outcome by influencing probability of conception, duration of stimulation, number of mature oocytes, cleavage, and fertilization and implantation rates. The cut off value of BMI for obesity (>30) according to WHO is different from (>26) in Asian population (41). The results of our study have documented positive outcome out of ARC treatment plans especially ICSI in women with BMI around 23-25.9 kg/m^2^ and lay emphasis on infertile women to maintain their body weight. 

## Conclusion

The results of our study concluded that overweight females with BMI 23-25.9 kg/m^2^, had maximum number of retrieved and fertilized oocytes which helped in blastocysts implantation and gestational sacs appearance on TVS. The oocyte recovery, cleavage, fertilization, implantation and clinical pregnancy rates were also higher in this group. It is thus imperative that in assisted reproductive clinics, women with BMI greater than 26 kg/m^2^ should be counseled and encouraged to reduce weight before treatment. 

This can only be made possible by counseling with evidence that extremes of BMI may negatively affect chances of successful conception after IVF treatment and pregnancy-related complications (18). The ﬁrst line of approach should be an emphasis through lifestyle modiﬁcation with careful counseling on selection of restricted calorie diet and involvement in program of exercise with sufficient aerobic activity. The outcome will be a sense of satisfaction as well as achievement for those who really care for the agonizing pains of infertile couples.

## Conflict of interest

There is no conflict of interest in this research.
